# The contribution of aestivating mosquitoes to the persistence of *Anopheles gambiae *in the Sahel

**DOI:** 10.1186/1475-2875-10-151

**Published:** 2011-06-06

**Authors:** Abdoulaye Adamou, Adama Dao, Seydou Timbine, Yaya Kassogué, Alpha Seydou Yaro, Moussa Diallo, Sékou F Traoré, Diana L Huestis, Tovi Lehmann

**Affiliations:** 1 Malaria Research and Training Center (MRTC)/Faculty of Medicine, Pharmacy and Odontostomatology, Bamako, Mali; 2 Laboratory of Malaria and Vector Research, NIAID, NIH. Rockville, MD, USA

## Abstract

**Background:**

Persistence of African anophelines throughout the long dry season (4-8 months) when no surface waters are available remains one of the enduring mysteries of medical entomology. Recent studies demonstrated that aestivation (summer diapause) is one mechanism that allows the African malaria mosquito, *Anopheles gambiae*, to persist in the Sahel. However, migration from distant localities - where reproduction continues year-round - might also be involved.

**Methods:**

To assess the contribution of aestivating adults to the buildup of populations in the subsequent wet season, two villages subjected to weekly pyrethrum sprays throughout the dry season were compared with two nearby villages, which were only monitored. If aestivating adults are the main source of the subsequent wet-season population, then the subsequent wet-season density in the treated villages will be lower than in the control villages. Moreover, since virtually only M-form *An. gambiae *are found during the dry season, the reduction should be specific to the M form, whereas no such difference is predicted for S-form *An. gambiae *or *Anopheles arabiensis*. On the other hand, if migrants arriving with the first rain are the main source, no differences between treated and control villages are expected across all members of the *An. gambiae *complex.

**Results:**

The wet-season density of the M form in treated villages was 30% lower than that in the control (P < 10^-4^, permutation test), whereas no significant differences were detected in the S form or *An*. *arabiensis*.

**Conclusions:**

These results support the hypothesis that the M form persist in the arid Sahel primarily by aestivation, whereas the S form and *An. arabiensis *rely on migration from distant locations. Implications for malaria control are discussed.

## Background

*Anopheles gambiae*, the principal malaria vector is a complex of species that occupies diverse habitats in sub-Saharan Africa including the dry Sahel [1-4]. The mechanisms that allow these species to survive the long dry season (4-8 months), when no surface waters are available, have been debated for over 70 years [5-12]. One explanation that has been proposed is that adults extend their survival during the dry season by undergoing aestivation (i.e. summer diapause) in (unknown) local shelters [13]. The alternative explanation proposes that migrants from distant locations, where permanent surface waters are available, colonize areas vacated by previous populations soon after the rains [12]. A recent study demonstrated that aestivation (summer diapause) is one mechanism that allows the M form of *An. gambiae *to persist in the Sahel [7]. Additionally, that study showed that very small numbers of *An. gambiae *adults, mostly fed and gravid females, can be found indoors (~0.035/house) throughout the dry season in the Sahel, presumably representing the larger hidden population of aestivating adults.

However, the authors could not rule out additional migrants from distant localities (>20 km), where reproduction continues year-round. While it is possible that migrants contribute more than aestivating adults to the persistence of populations in the Sahel, records of movements of *An. gambiae *beyond 2 km in distance are rare [14,15] and no report exceeds 10 km. Knowledge of the source of the early wet season population in arid and semi-arid habitats represents a critical gap in our understanding of this vector's ecology. This knowledge may have important implications for vector and malaria control.

This study was undertaken to assess the relative contribution of aestivating adults versus migrants to the buildup of Sahelian anopheline populations in the subsequent wet season. Specific predictions of the aestivation and migration hypotheses were tested. Thus, on the one hand, if the primary source of the mosquitoes that seed the new wet-season populations consists of locally aestivating adults, then reducing the population during the dry season by weekly indoor spray with a short-duration insecticide such as pyrethrum will reduce their survival and impact the buildup of the wet season population. On the other hand, if migrants from distant locations play the key role, then the reduction of the local population during the dry season will have a negligible effect on the buildup of the wet-season population. Density during the dry season at this Sahelian area remains very low until 3-6 days after the first rain, when numbers surged over ten-fold [7]. If these mosquitoes represent migrants, they arrive just after the first rain and the impact of the treatment (pyrethrum effect lasts only 1-2 days after application) on them would be minimal. If migrants arrive before the first rain, they should be detected by our monitoring. Accordingly, it was predicted that under the aestivation hypothesis, the treatment should reduce population density in treated villages as opposed to untreated control villages (Density**_T _**<Density**_C_**). Importantly, this prediction applies exclusively to the M molecular form of *An. gambiae*, but not to the S form or *Anopheles arabiensis *because the latter taxa are not found indoors during the dry season [7] and thus cannot be affected by the treatment. On the other hand, if mosquitoes migrate from outside the area to establish the new wet-season population, then this treatment will have little effect on the migrants and therefore (Density**_T _**= Density**_C_**) for all members of the *An. gambiae *complex, including the M form.

## Methods

**Figure 1:**
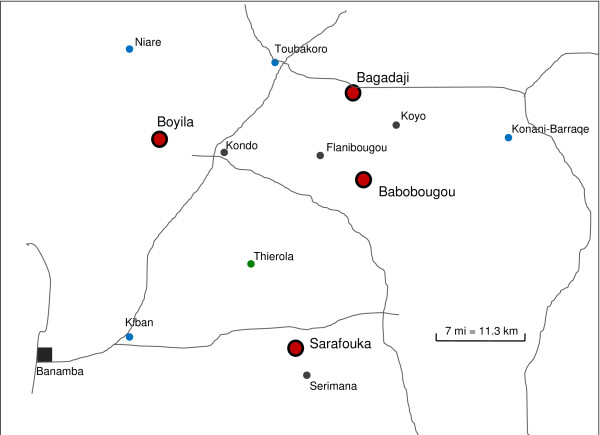
**A schematic map showing locations of the four focal villages (red large dots)**. The nearest village (gray dots) and nearest permanent surface waters (blue dots) to each of the focal villages are shown as well as the site of a previous study (Thierola), to which several citations were made. Roads (unpaved) are shown in gray and the largest town (Banamba) is marked as a gray square. Additional information is provided in Table 1.

Four villages (Table 1 and Figure 1) were selected for this study based on the following criteria. Each selected village was required to be located in the Sahel, over 3 km away from the nearest village to minimize migration from neighbouring villages, and at least 10 km from any larval site that remained with water after January to ensure that no local mosquito reproduction was possible during the treatment period (see below). Only villages of small to medium population size were selected, so all houses (including kitchens, household storage, chicken houses, etc.) can be sprayed in a single day. Selected villages were divided into pairs based on proximity to each other and villages in each pair were randomly assigned to either the treatment or control village group. Weekly treatment of pyrethrum spray in all houses of treated villages started after the desiccation of the last larval site in a radius of 10 km around each village and continued until the first rain. The first treatment was applied on December 20, 2009 and the last one on May 23, 2010. In the other two villages (controls), only monthly monitoring was performed. Monitoring consisted of indoor pyrethrum spray in the same 25 houses in each village, performed on the same day in each treatment-control village pair and on consecutive or nearly-consecutive days on the other pair. Monitoring of population size and species composition was conducted monthly in each village from September 2009 to mid-May 2010 and approximately every ten days from mid-May until the end of October 2010. The effect of monitoring on mosquito populations of the control villages was small (and conservative) because it consisted of once-a-month spray of 25 houses of 188 and 195 houses in total (Table 1) rather than once-a-week spray of all houses as was done in the treated villages during the treatment. This increased frequency of monitoring should enhance the resolution of the comparison of the buildup of the wet-season populations in the treated and untreated (control) villages. Collected mosquitoes were identified visually to separate *An. gambiae *s.l from other species and later subjected to the genetic identification of the sibling species and molecular forms as previously described [16].

**Table 1:** Village information

*Village^a^*	*Coordinates*	*Houses^b^*	*Pair^c^*	*Near Village^d^*	*Near Water^e^*
Babobougou (T)	13 45N,7 04W	58	1	7	17
Boyila (T)	13 47N,7 20W	198	2	9	17
Sanafouka (C)	13 33N,7 10W	188	1	3	22
Bagadaji (C)	13 51N,7 06W	195	2	7	11

^a ^Treated villages (T) and control villages (C).

^b ^Number of houses in the village.

^c ^Pair number. The distance between treated and control village in each pair was 25 km.

^d ^Distance (km) to the nearest village.

^e ^Distance (km) to the nearest body of surface water which holds water after December.

### Statistical analysis

To detect heterogeneity in species and molecular form composition between samples, exact tests were performed on contingency tables using Proc Freq in SAS 9.2 (SAS Institute, Inc., Cary, NC) [17]. Sequential samples from the same village were pooled only if the sample size of one (or both) was small (N<30, as was the case during parts of the dry season, e.g., February and March) and there was no significant heterogeneity in species and/or form composition between them. Global tests were employed to evaluate significance of multiple tests. The sequential Bonferroni procedure [18] was used to test individual departures from the null hypothesis, such as non-homogenous (heterogeneous) species composition in one village during a particular sampling period. The binomial test (which estimates the probability of obtaining the observed number of significant tests at the 0.05 level given the total number of tests) was used to detect weaker departures across multiple tests.

To compare densities in treated and control villages over the series of sampling time periods after the first rain, the series of the differences (Treated-Control) were calculated by subtracting the corresponding values of the same sampling period. A permutation test was used because the values of a time series are serially correlated, and the series of differences may have retained that effect. Accordingly, density values were randomly assigned to either the treated or control village (stratified by village pair) prior to calculating the difference between the "Treated" and "Control" values for each time point. The distribution of mean difference derived from each of the 10,000 pseudo-samples provided the basis to determine if the observed mean of the series of differences is smaller than random expectations for each species and molecular form (see hypotheses, above). Initially, a global test (across villages comparing treated and control villages) was used for each of the three taxons. If the global test was insignificant (P > 0.05), the decision was reached and no additional tests were used. However, if the global test was significant (P < 0.05), the same test was applied to compare the treated and control villages in each village pair for the significant taxon.

## Results

A total of 17,430 An. gambiae s.l. (10,602 females and 6,828 males) were collected from the four villages over 22 sampling periods using pyrethrum knock-down in 25 houses/village. Identification to species and molecular form was performed on 4734 *An. gambiae *s.l. (3811 females and 853 males) of which 73.0% were M-form *An. gambiae*, 15.1% were S form and 8.6% were *An. arabiensis *(3.2% of the mosquitoes could not be identified due to poor preservation of DNA and 0.1% (n = 3) were M/S hybrids).

**Figure 2:**
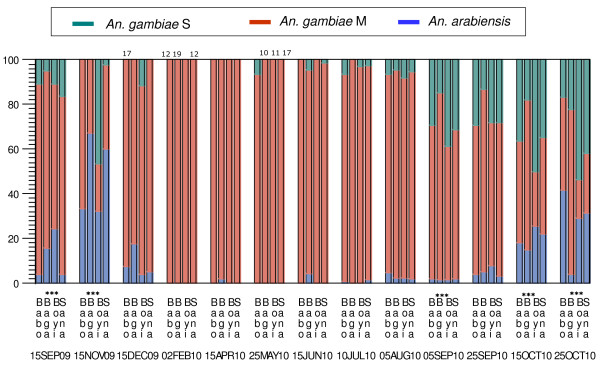
**Species and molecular form composition in the four villages over time, based on 4,584 mosquitoes which were successfully genotyped to species and molecular form (see text for details)**. Note that the time intervals are variable. Pooling of adjacent dates was carried out if samples were small and only if minimal differences in composition were found. Except for the period marked '2Feb10' that covered samples from January to March, other pooled samples represent periods shorter than three weeks between June and October 2010. Numbers above bars show cases where sample size per village/period were smaller than 20. Significant heterogeneity among villages (P < 0.05, after the sequential Bonferroni procedure) is denoted by stars.

The species and molecular form composition did not vary between males and females because only three of 49 tests indicated heterogeneity (individual tests: 0.0021 < P < 0.05; Binomial multi-sample test: P < 0.44). None of these tests were significant at the multi-test level, using the sequential Bonferroni procedure. Therefore, in all subsequent analyses males and females collected at the same sampling period were pooled. Variation in species and molecular form composition was detected among villages with eight significant tests (individual tests: 10^-5 ^< P < 0.05) of 16 tests (Binomial multi-sample test: P < 0.001). All significant tests (at the multi-test level using the Bonferroni procedure) were clustered in the late wet season: September-October 2009 and from the August to October 2010. Overall, the seasonal variation in species and molecular form composition (Figure 2) followed the pattern previously reported for this region [7].

**Figure 3:**
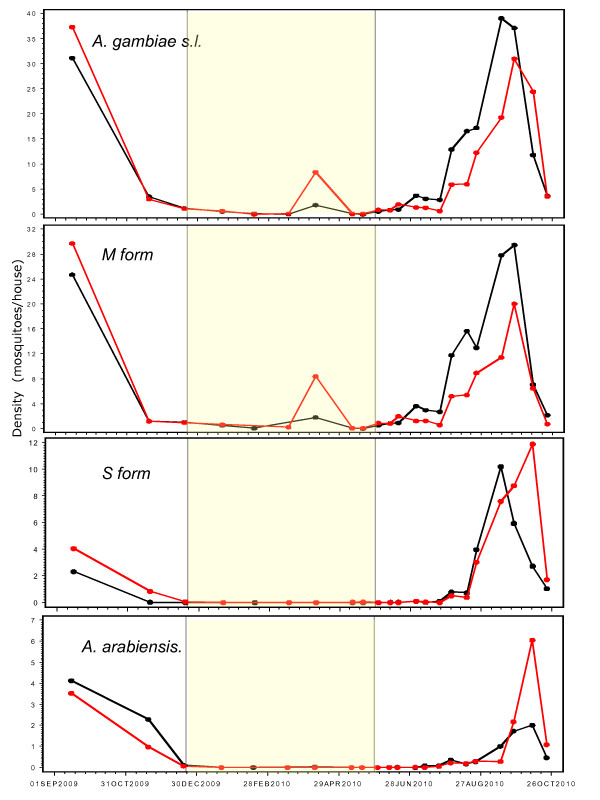
**Overall density (number of mosquitoes/house) in treated (red) and control (black) villages over time, measured by pyrethrum spray collections in 25 houses/village every month until the first rain and every 10 d thereafter**. The density of the molecular forms of *An. gambiae *and of *An. arabiensis *was estimated by multiplying the density of *An. gambiae *s.l. (upper panel) by the corresponding fraction representing the relevant taxon in the corresponding village and time period. The yellow shading denotes the period of treatment in treated villages (from the desiccation of the last larval site 10 km around the village and until the first rain).

**Figure 4:**
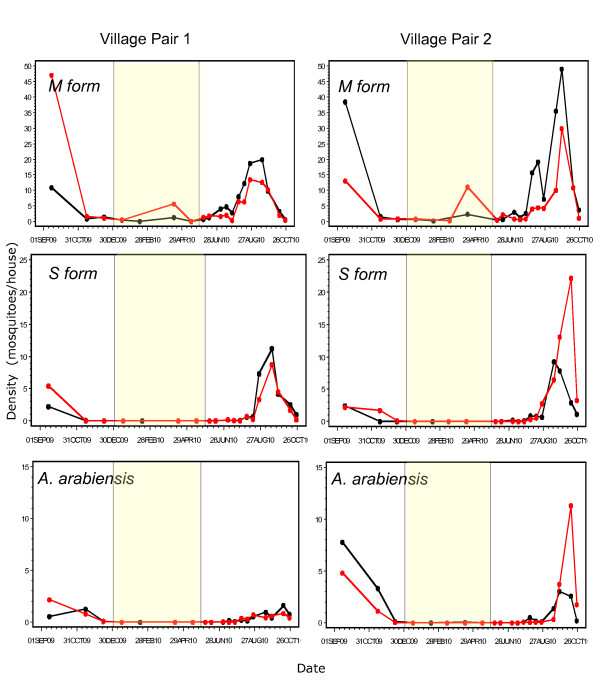
**Density (number of mosquitoes/house) in each pair of treated (red) and control (black) villages over time (for further details, see legend of Figure 3)**.

Density in the four villages was monitored starting in the late wet season (September 2009). During that period, overall density was high (approx. 30/house, range: 14-55; Figure 3) and the species and molecular form composition changed rapidly (Figure 2) as previously reported for that region [7]. At that time, overall density in the villages selected for treatment was higher or equal to that of the control villages (Figure 3). However, vector composition and density varied among villages and even between paired villages (Figures 3 and 4) similar to previous reports, e.g. [4].

The last larval site dried up in December, almost two months after the last rain (October), when the average density dropped to 1.1/house (range 0.7-1.5; Figure 3) and treatment started (in treated villages). Over the next three months, the average density of *An. gambiae *s.l. continued to drop reaching 0.05-0.07/house (range: 0-0.24) by February and March. This low density lasted until May, except during 3-5 days in April, when mosquito density surged (April 8-10) across the region, which happened to coincide with the monitoring date (Figures 3 and 4). Remarkably, the density returned to its typical low dry-season density from April 11^th ^(In Preparation). Despite the absence of surface water for larval sites and regardless of weekly insecticide treatments, mosquitoes were present (albeit at low densities) in all villages throughout the dry season. During the dry season (Dec-May), the M form represented >95% of the individuals (>99% from January-May, Figure 2).

The first rain (31 mm) fell on May 29, 2010, filling empty larval sites with enough water to last over 10 d. The last weekly pyrethrum spray was performed on May 19 (Babobougou) and on May 23 (Boyila). Three days after the first rain (before reproduction could increase adult population sizes), average density increased 12.9 fold, from an average of 0.055/house (range: 0-0.12) earlier in May to 0.71/house (range: 0.4-1.32). The mosquito density continued to increase until October, however, the average rate of increase over ~10 d intervals during this period was 1.73 (range: 0.8-5.4). During May and June, composition remained dominated by the M form (>97%), although a single *An. arabiensis *was found in mid-June and two S-form mosquitoes were found in mid-May, before the first rain (Figure 2).

The overall effect of the treatment was measured as the difference between treated and control villages over time, during the entire wet season (June to October 2010, see Methods). Population density in treated villages was 30% lower than in control villages (Table 2) as predicted based on the aestivation hypothesis. A significant difference was detected exclusively in the M molecular form (Table 2, Figures 3 and 4). The effect on the M molecular form was not only detected in the global test (across village pairs), but also in each village pair separately (Table 2).

**Table 2:** The effect of treatment on the buildup of wet-season populations

Taxon	Density_T-C_^a^	P	Village Pair 1	P	Village Pair 2	P
***A. gambiae *M form**	**-4.9 (-30%)**	**P < 10^-4^**	**-2.5 (33%)**	**0.002**	**-7.2 (27%)**	**<10^-4^**
*A. gambiae *S form	0.7 (19%)	P > 0.29	ND ^b^		ND ^b^	
*A. arabiensis*	0.4 (42%)	P > 0.71	ND ^b^		ND ^b^	

^a ^The average difference in mosquito density between treated and control villages across all time points throughout the rainy season (June to October). In parenthesis, the relative change in the treated villages with respect to the control expressed as a percentage from the control.

^b ^ND denotes not determined because global test was insignificant (see Materials and Methods.

## Discussion

The contribution of aestivating mosquitoes to the persistence of anopheline malaria vectors in Sahelian villages was evaluated in this study. If aestivating mosquitoes that periodically blood feed (albeit less frequently) constitute the main source of the population after the 6-7 months long dry season [7,13,19] then weekly pyrethrum sprays during the dry season would reduce this source, resulting in smaller wet-season populations. On the other hand, if migrants from distant locations constitute the main source [12,20,21], they would not be affected by this treatment and no reduction in density can be expected, unless these migrants arrive before the first rain. If migrants arrive to the area, they probably arrive after the rains because density increased over tenfold 3-5 d after the first rain ([7] and this study). Formulating separate predictions for each species and molecular form further increased the stringency of this multi-hypothesis test. Our previous study showed that the M form is found (indoors) almost exclusively in this Sahelian area during the dry season [7]; therefore it was predicted that the treatment should not affect the S molecular form and *An. arabiensis*. Indeed as per our *a priori *expectations, the results showed a significant reduction in wet-season populations of the M molecular form in treated vs. control villages, whilst no such effect was detected in the case of either the S form or *An. arabiensis*. These results are highly consistent with the predictions under the aeastivation hypothesis and are not consistent with the migration hypothesis (see below). This is strong evidence that aestivation is a major mechanism used by the M form to persist throughout the dry season in the Sahel away from permanent surface water.

Surprisingly, mosquito density rose dramatically across our study area for 3-5 days in the midst of the dry season (April 7-10, 2010). This surge might represent the arrival of migrants from distant location(s) or the appearance of locally aestivating mosquitoes that have been hidden in (unknown) shelters nearby. If this surge reflects the arrival of migrants prior to the first rain, then such migrants could be affected by our treatment and the difference between treated and control villages may be equally explained by migration. However, if these were the migrants that were to establish the subsequent wet season, it is difficult to explain their disappearance shortly after they peaked. Importantly, density later in April and throughout May was as low as that prevailing in February and March across the area (Figures 3 and 4). These putative migrants might have continued their migration elsewhere or died shortly after arriving, because no trace of elevated density remained in the area. Had these migrants been the seed of the next wet-season population, they must have hidden in shelters, as aestivating mosquitoes presumably do. Moreover, they would have to survive over eight weeks until the first rain. Typical wet-season adult *An. gambiae *only survive up to 3-4 weeks [14,15,22,23], even if they have not migrated long distances. Survival of eight weeks has never been recorded for *An. gambiae *except under aestivation [7,13,19]. Thus, if the elevated April density was due to migrant mosquitoes, these would have to be capable of both migration and aestivation (seemingly rendering the migration pointless). Finally, the source of these putative migrants remains elusive. The density of the source must be unimaginably high to supply so many migrants to the hundreds of Sahelian villages in the region, because total village density in each of the six villages that was examined (Thierola, M'piabougou, and the four focal villages of this study) were in the thousands. No locality (known to us) in over a hundred km radius around the study area had normal dry-season density equal to that found during the surge, let alone one with an even higher density. In summary, arguing that the surge of mosquitoes during early April represents migrants that formed the early wet-season population cannot explain (i) their rapid disappearance, especially from untreated villages, (ii) their survival through the 8-week long period between the surge and the first rain, and (iii) the absence of a plausible "source" at least up to a 100 km away. Therefore, it is concluded that the mosquitoes that surged in early April are unlikely to represent migrants that established the subsequent wet-season population. On the other hand, the possibility that these mosquitoes represent locally-aestivating adults that emerged from their shelters to replenish nutritional reserves and returned to shelters several days later is consistent with everything known and presumed about aestivation, except for its synchrony. Therefore, this observation is more consistent with aestivation than with migration.

Unlike the M form of *An. gambiae*, our results suggest that the mechanism of persistence for the S form and *An. arabiensis *does not involve aestivation. Possibly, these taxa do not feed and rest indoors throughout the dry season and thus may not respond to our treatment. However, while the M-form density increased over tenfold within three days after the first rain and continued to increase at a slower rate afterwards, the density of the S form and *An. arabiensis *remained stable and virtually zero for over four weeks after the first rain (Figures 2, 3, and 4). Had aestivating adults of the S form and *An. arabiensis *survived throughout the dry season, it is unlikely that they would not emerge for over four weeks after the rain filled all larval sites. These results suggest that both the S form and *An. arabiensis *perists in the Sahel by migration from distant locations.

The reduction in the wet-season density of the M form in treated villages was moderate (30%, Table 2), indicating that our treatment killed only a fraction of the aestivating population. This is consistent with the short duration of pyrethrum lethality [24] and with the presumption that during the dry season mosquitoes spend most of their time in unknown hidden shelter [7]. Further, the evaluation of the treatment's effect is conservative because it encompasses nearly the full rainy season although the effect diminished with time after treatment was stopped. Finally, reduced peak population size during the Sahelian rainy season suggests that the number of generations during population growth was not large enough to overwhelm the size of the founding population.

How many mosquitoes enter aestivation in an average Sahelian village and how many of them survive until after the first rains? Knowledge of the dynamics of aestivating populations may be key for vector control programs targeting this critical phase of the vector. Exact estimates are beyond our reach, but estimates of the minimum number of mosquitoes that enter and complete aestivation may be derived from our results. Multiplying the mosquito density/house throughout the treatment period by the number of houses in each treated village and the number of treatments/month, the total *An. gambiae *s.l. and M-form mosquitoes that were killed in each village were estimated (Table 3). Likewise, the minimum number of mosquitoes that presumably completed aestivation was estimated as the product of the house density 3-6 d after the first rain multiplied by the total number of houses in each village (Table 3). Accordingly, the minimum number of mosquitoes that completed aestivation is estimated near 100 whereas the estimate of the minimum number that entered aestivation varied between 500 and 3,000 (Table 3). Note that these are estimates of the minimal values whereas the actual values may be substantially larger.

**Table 3:** Estimates of the minimum numbers of mosquitoes impacted by the treatment, surviving until after the first rains, and the derived estimate of the minimum number that entered aestivation

*Village ^a^*	*Houses ^b^*	*Dry Killed ^c^*	*EarlyWet ^d^*	*EnterAest ^e^*
Babobougou (T)	58	469	77	546
Boyila (T)	198	2,965	87	3,052
Sanafouka (C)	188	44	128	ND^f^
Bagadaji (C)	195	78	78	ND^f^

^a ^In treated villages (T), all houses were subjected to insecticide application 4 times per

month, whereas in control villages (C), only 25 houses were subjected to insecticide application once per month.

^b ^Total houses in the village, including houses used as kitchens, storage, etc.

^c ^Estimate of the total *An. gambiae *M form that were killed by treatments, calculated as the sum over months (January - May) of the product of the M form monthly house density multiplied by total number of treated houses and the number of treatments per month. In treated villages during April, the high density was used to calculate the number of mosquitoes killed in one treatment and the average across February, March, and May was used for the other three treatments.

^d ^Estimate of the total *An. gambiae *M form that were present 3-6 d after the first rain, calculated as the product of the M form house density at that time multiplied by total number of houses in the village.

^e ^Estimate of the minimal *An. gambiae *M form that entered aestivation in each village, calculated as the sum of the those killed throughout the dry season and those that survived until after the rains.

^f ^This was not determined (ND) for the control villages.

Could a malaria control program targeting aestivating mosquitoes in Sahelian villages be effective? Coupling long-lasting insecticides to extend the treatment throughout the dry season and during the early emergence of aestivating adults from their shelter is expected to dramatically increase the efficiency of the treatment in reducing populations of the M form. However, peak malaria transmission occurs in September-October [25], when the S molecular form and *An. arabiensis *increase in density and often predominate (Figures 2 and 3; [7]).

Because they are not affected by the treatment during the dry season and presumably do not aestivate (unlike the M form; see above), it appears that targeting aestivating mosquitoes may have a limited effect on the malaria burden. Nevertheless, malaria transmission requires successive amplification "cycles", whereby vectors are infected and transmit "back" into new human hosts, which in turn increases mosquito infection, and so on. Targeting aestivating mosquitoes may cut these early amplification cycles [26]. Since the mosquito season (and subsequently the malaria transmission season) is bound by the rains, reducing the magnitude of the early cycles might have strong exponential impact on malaria transmission, with peak transmission intensity in September-October reduced to the same levels as in July-August. Continued treatment for several years may further benefit malaria control by the cumulative effect of a reduced human reservoir. Therefore, it is proposed here that targeting aestivating mosquitoes in the dry season could have immense public health benefits for communities living in the Sahel and in other arid areas where surface waters are absent for over three months.

## Conclusions

These results support the hypothesis that aestivating M-form *An. gambiae *mosquitoes contribute substantially to the persistence of their populations in arid Sahelian regions whereas the S form and *An. arabiensis *rely on migration. This study provides fresh insight into one of the most enigmatic and oldest problems of medical entomology. Because malaria transmission in the Sahel occurs typically over a short period of three months, targeting aestivating mosquitoes could reduce the early "rounds" of vector-human amplifications and potentially reduce malaria transmission exponentially.

## Competing interests

The authors declare that they have no competing interests.

## Authors' contributions

TL conceived of the study, participated in its design and coordination, performed the statistical analysis, and helped to draft the manuscript. AA, AD, ASY, and SFT participated in the design of the study. AA led and carried out the field work with ST, AD, ASY, MD, and YK. YK led and carried out the genotyping with AA ST, and DLH. DLH and AA participated in drafting and revising the ms. All authors read and approved the final manuscript.
